# Data on the effect of in utero exposure to polycyclic aromatic hydrocarbons on genome-wide patterns of DNA methylation in lung tissues

**DOI:** 10.1016/j.dib.2017.06.023

**Published:** 2017-06-21

**Authors:** Trevor J. Fish, Abby D. Benninghoff

**Affiliations:** a Department of Animal, Dairy and Veterinary Sciences, Utah State University, Logan, UT 84322, USA; b School of Veterinary Medicine, Utah State University, Logan, UT 84322, USA

## Abstract

Data in this article depict patterns of methylation in lung tissues obtained from the offspring of B6129SF1/J dams and 129S1/SvImJ sires exposed *in utero* to benzo[*a*]pyrene (BaP) or dibenzo[*def,p*]chrysene (DBC) as compared to non-exposed offspring. Genome-wide methylation of lung tumors in adult offspring was determined using methylated DNA immunoprecipitation (MeDIP) with the NimbleGen mouse DNA methylation CpG island array. This data article refers to the research article “DNA methylation in lung tissues of mouse offspring exposed *in utero* to polycyclic aromatic hydrocarbons,” [1] in which comprehensive data interpretation and analysis are provided.

## **Specifications Table**

**Table:** 

Subject area	*Molecular Biology*
More specific subject area	*Epigenetics, Toxicology*
Type of data	*Excel spreadsheets, figures and tables*
How data was acquired	*Methylated DNA immunoprecipitation (MeDIP) with NimbleGen mouse DNA methylation CpG island array*
Data format	*Raw, filtered and analyzed*
Experimental factors	*Described in the text*
Experimental features	*Very brief experimental description*
Data source location	*Utah State University, Logan, Utah, United States of America*
Data accessibility	*Data is available in public repository or within this article*
Related research article	*Fish, T.J. and A.D. Benninghoff. (In press) DNA methylation in lung tissues of mouse offspring exposed in utero to polycyclic aromatic hydrocarbons. Food Chem Toxicol.*

## **Value of the data**

• Data provide profiles for genome-wide DNA methylation for normal lung tissue, normal-adjacent lung tissue and tumor lung tissue from mice initiated with model polycyclic aromatic hydrocarbons (PAH) *in vivo*.
• Ontology analysis revealed biological processes associated with differentially methylated genes in normal or tumor tissues.
• Data may be mined to identify biomarkers of *in utero* PAH exposure or compared to patterns of DNA methylation in lung tissues for other exposures to environmental toxins.

## 1 Data

### 1.1 NimbleGen processed data report for all or nearest methylated peaks

The processed data sets obtained using the NimbleGen Mouse DNA Methylation 3×720K CpG Island Plus RefSeq Promoter Array are provided as archived Excel files, including all peaks (Supplementary File 1; see DOI referenced in Fish [2] ) and peaks nearest to the transcription start site (Supplementary File 2; see DOI referenced in Fish [3]). Each.zip archive includes 15 individual documents, one for each sample hybridized to the NimbleGen mouse methylation array. File names reference each sample type and are provided in the accompanying readme document. Also, included in the readme document is a description of the file content according to the spreadsheet column title.

### 1.2 Peak scores summary table for all methylated genes in any data set

A summary table of peak scores for the nearest peak to the indicated transcript for any peak significantly methylated (score≥2.0) in any of the 15 samples analyzed are provided as a Microsoft Excel file available in the following reference (Supplementary File 3; Fish [4]). An accompanying readme file provides a description of the file content according to the spreadsheet column header.

### 1.3 Hierarchical clustering analysis

**Fig. 1:**
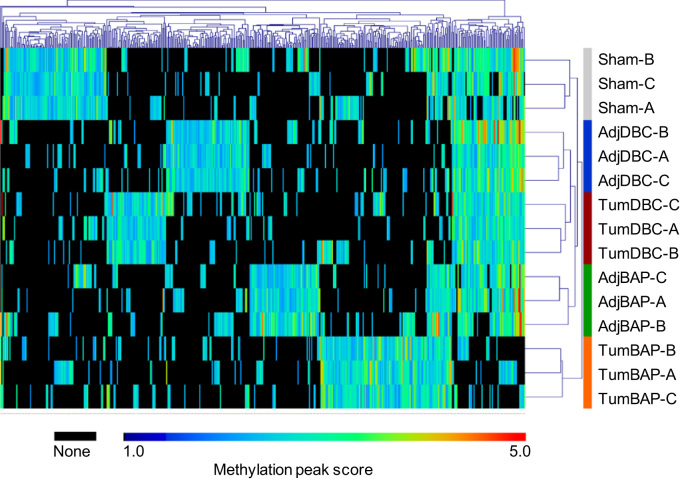
Unsupervised, bi-directional hierarchical cluster analysis for genes differentially methylated among sample types. Clustering was performed using peak score values, indicated by the color scale. Black indicates no apparent methylation as detected by the NimbleGen Mouse DNA Methylation 3×720K CpG Island Plus RefSeq Promoter Array (gene not listed in processed nearest peak data set) (Supplementary File 3; Fish [4]).

Fig. 1 depicts results of unsupervised, bi-directional hierarchical clustering analysis of genes differentially methylated among sample types, following criteria described below in Section 2.3.

### 3.4 Gene lists for ontology analyses

Table 1 indicates the specific comparisons performed to generate gene lists for ontology analysis and the number of genes in each resulting data set. These lists are provided as a Microsoft Excel file available at the DOI link included in the following reference (Supplementary File 4; Fish [5]). Within this spreadsheet, each column contains a set of gene accession numbers (MGI accession) representing genes methylated in one (or more) tissue type(s) and not in others according to the comparisons outlined in Table 1.

**Table 1:** List of comparisons among tissue types to generate gene lists for ontology analyses.

Comparisons	Number of genes in list
Methylated in Sham but not AdjDBC or TumDBC	271
Methylated in AdjDBC but not Sham or TumDBC	147
Methylated in TumDBC but not Sham or AdjDBC	150
Methylated in Sham but not AdjBAP or TumBAP	160
Methylated in AdjBAP but not Sham or TumBAP	159
Methylated in TumBAP but not Sham or AdjBAP	218
Methylated in Sham but not TumDBC or TumBAP	239
Methylated in TumDBC but not Sham or TumBAP	127
Methylated in TumBAP but not Sham or TumDBC	143
Methylated in TumDBC and TumBAP but not Sham	93

### 1.5 Results of gene ontology analyses for differentially methylated genes

Gene ontology was performed using AgriGO [6] using the singular enrichment analysis (SEA) tool against the mouse gene ontology database (Mouse Genome Informatics) as described in more detail below. The Microsoft Excel document available at the DOI link included in the following reference (Supplementary File 5; Fish [7]), contains three spreadsheets with GO terms for biological process (P), molecular function (F) and cellular component (C) for each of the comparisons outlined in Table 1 above, organized as follows:

• Sheet 1. AgriGO GO Slim Results for Biological Process, Molecular Function and Cellular Compartment for Sham, AdjDBC and TumDBC Tissues
• Sheet 2. AgriGO GO Slim Results for Biological Process, Molecular Function and Cellular Compartment for Sham, AdjBaP and TumBaP Tissues
• Sheet 3. AgriGO GO Slim Results for Biological Process, Molecular Function and Cellular Compartment for sham, TumDBC and TumBaP Tissues

Values shown are the *p* value for term enrichment using the Fisher test with false discovery rate (FDR) under dependency correction and the minimum number of mapping entries set at 5 genes.

### 1.6 Gene ontology maps

Gene ontology maps were generated for all group comparisons outlined in Table 1. These maps are provided as high resolution .tif files at the DOI link available in the following reference (Supplementary File 6; Fish [8]). This archive includes 10 individual image files, each of which depicts a gene ontology map for GO terms representing hypermethylated gene promoters unique for the indicated tissue(s) compared to other tissues.

### 1.7 Aligned signal map gene-specific methylation profiles

**Fig. 2:**
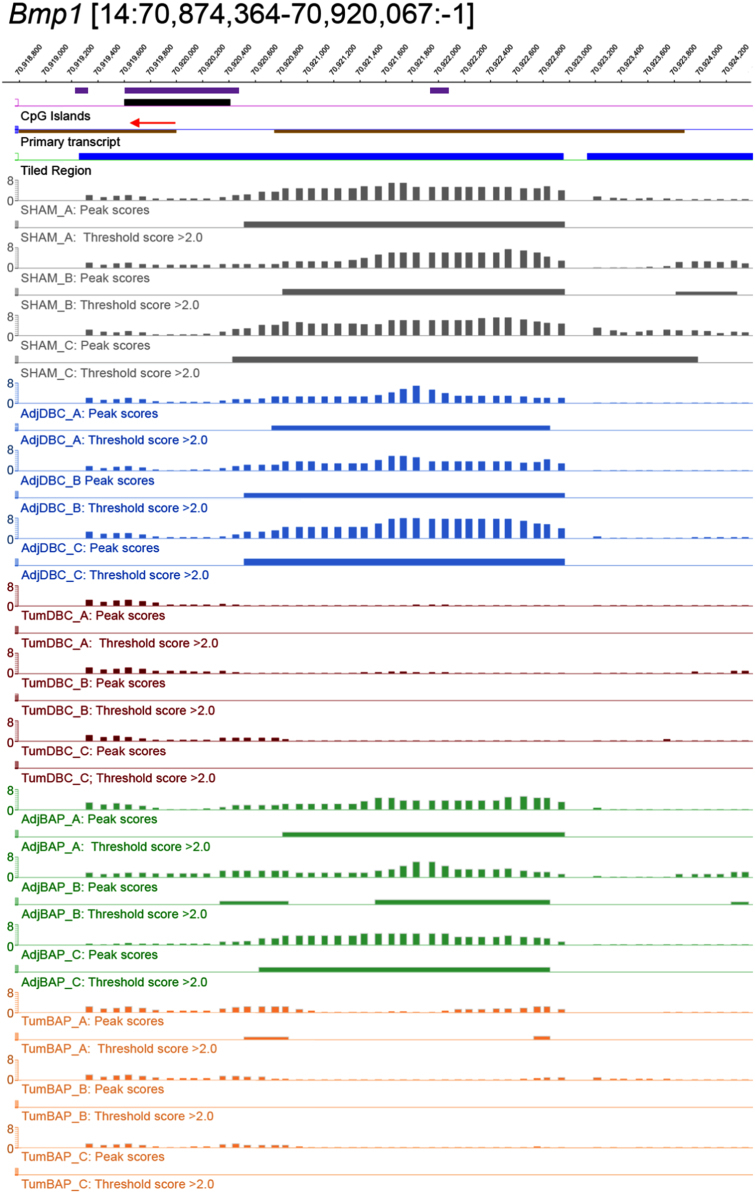
Methylation of *Bmp1* promoter in normal and tumor lung tissues.

**Fig. 3:**
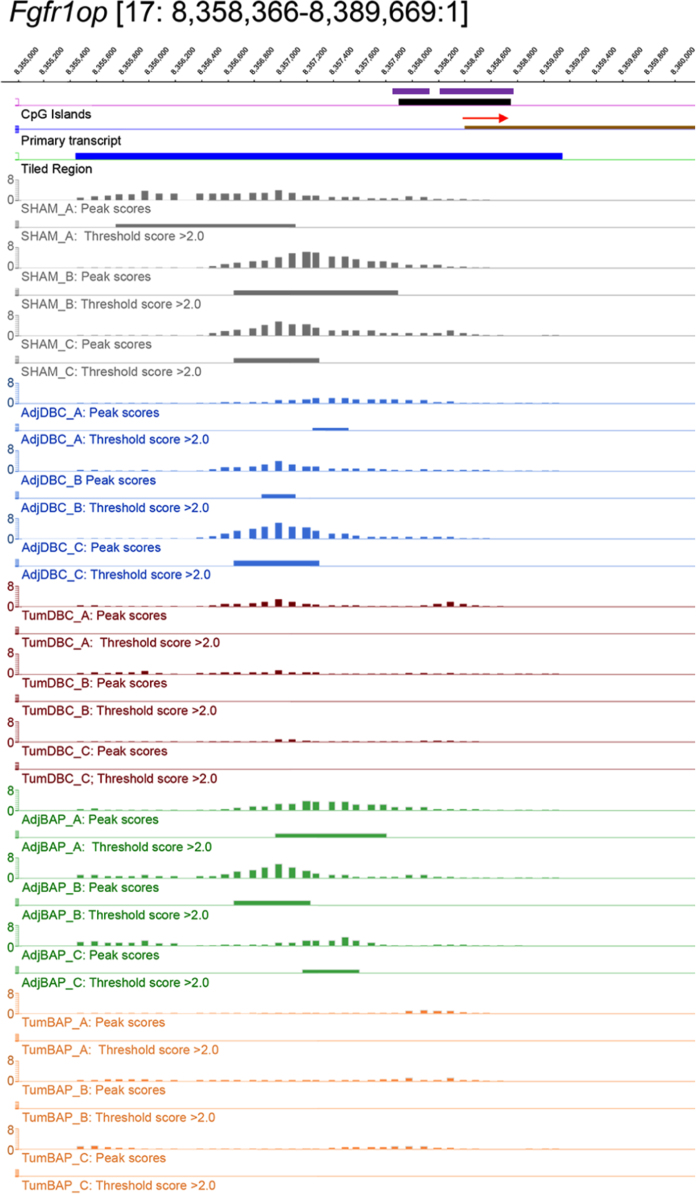
Methylation of *Fgfr1op* promoter in normal and tumor lung tissues.

**Fig. 4:**
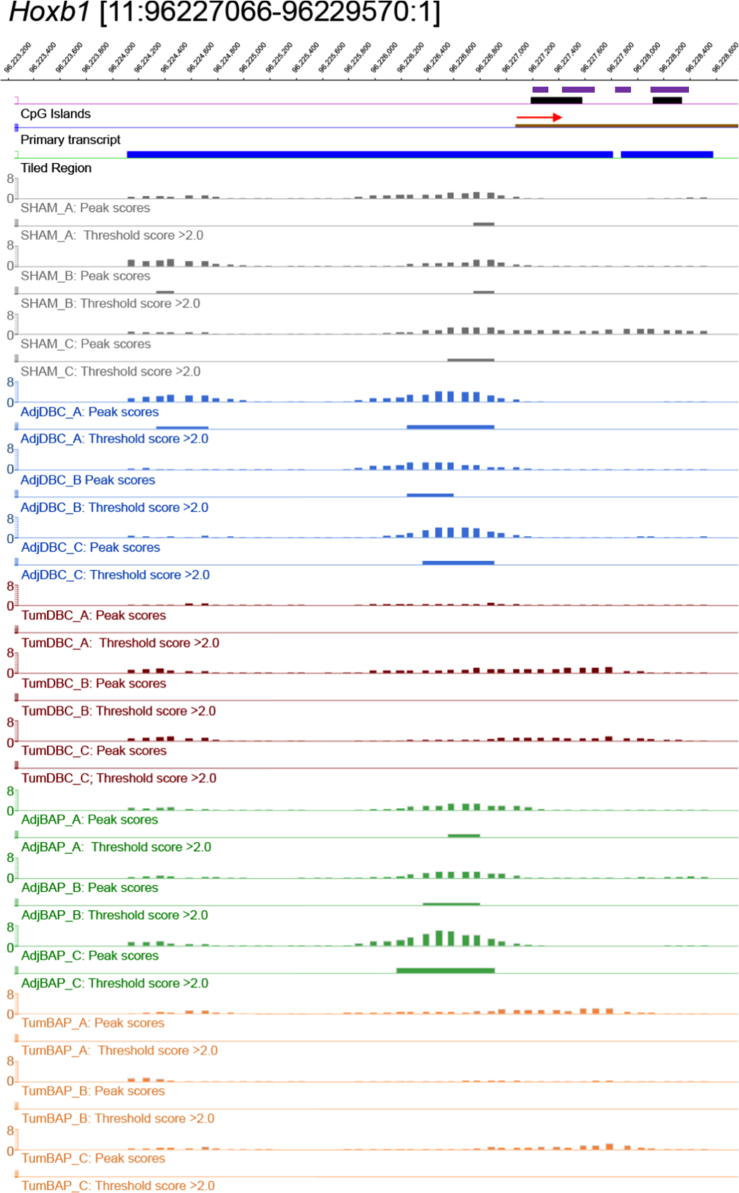
Methylation of *Hoxb1* promoter in normal and tumor lung tissues.

**Fig. 5:**
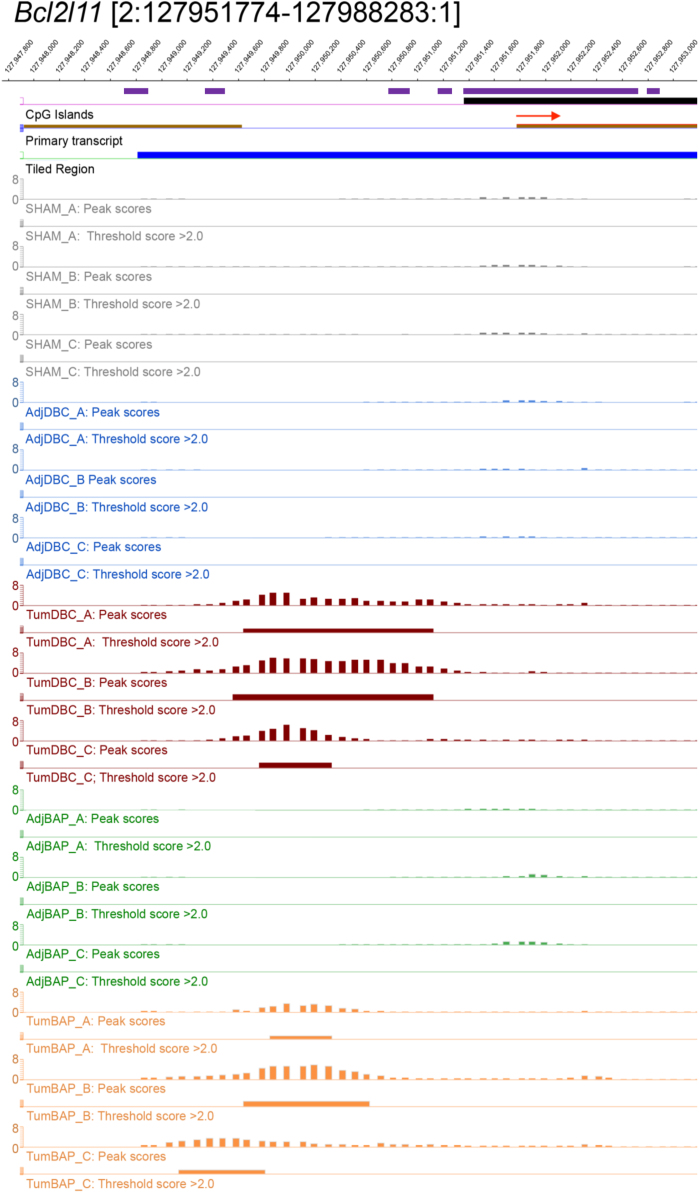
Methylation of *Bcl2l11* promoter in normal and tumor lung tissues.

**Fig. 6:**
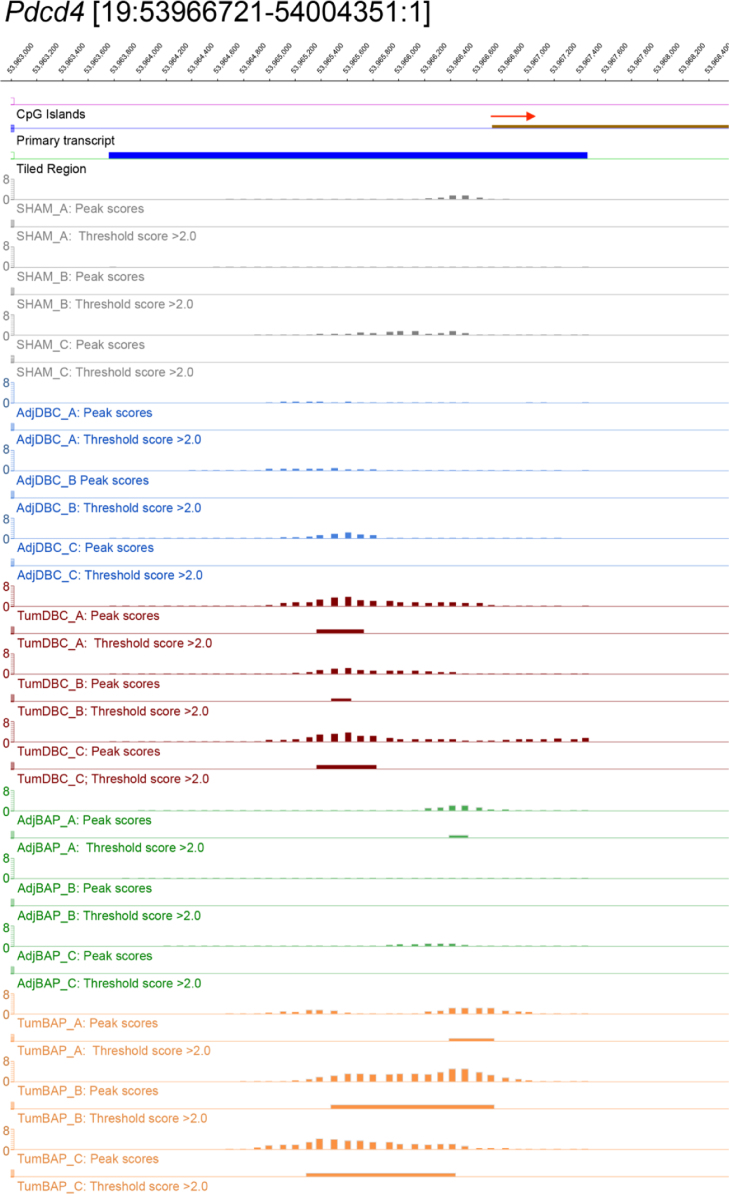
Methylation of *Pdcd4* promoter in normal and tumor lung tissues.

**Fig. 7:**
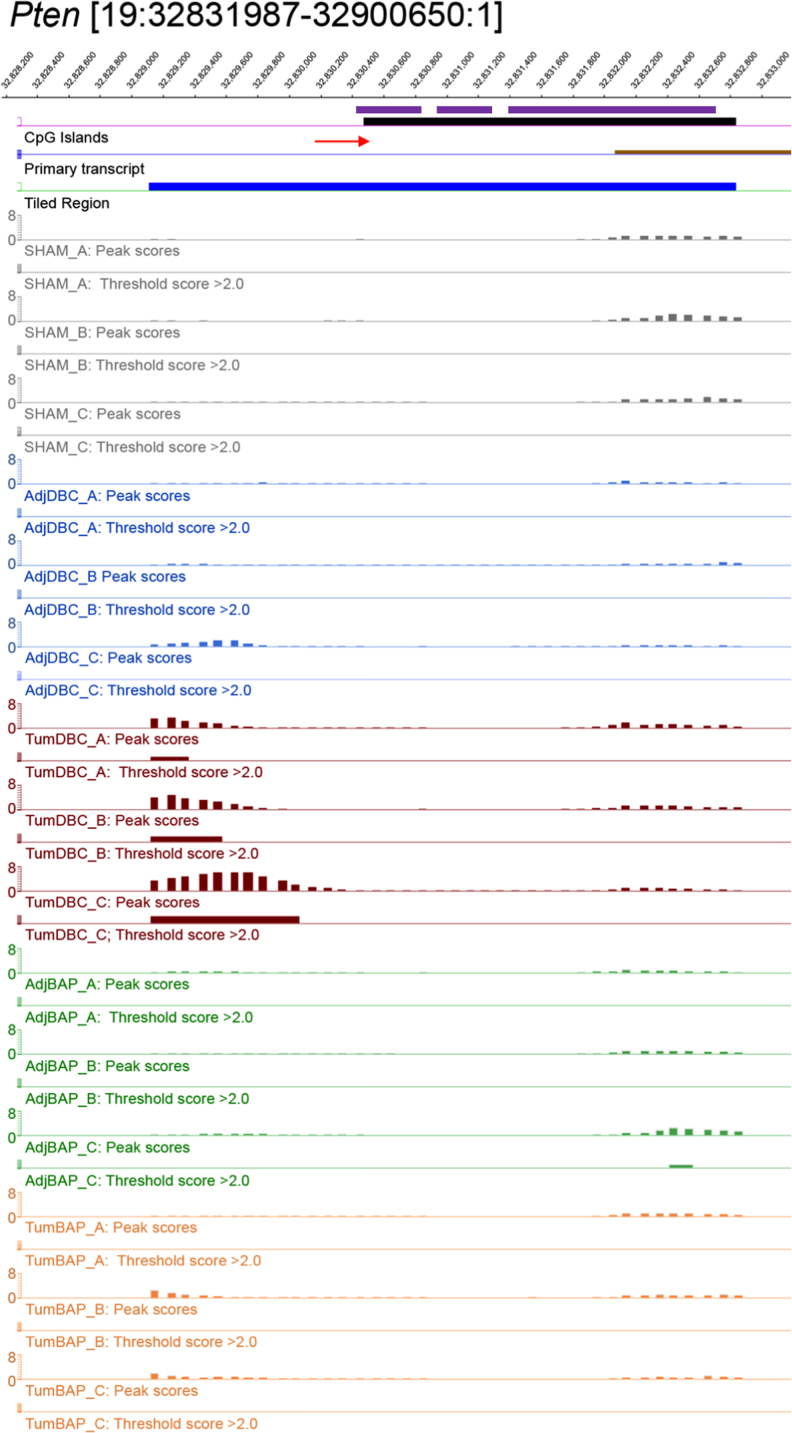
Methylation of *Pten* promoter in normal and tumor lung tissues.

**Fig. 8:**
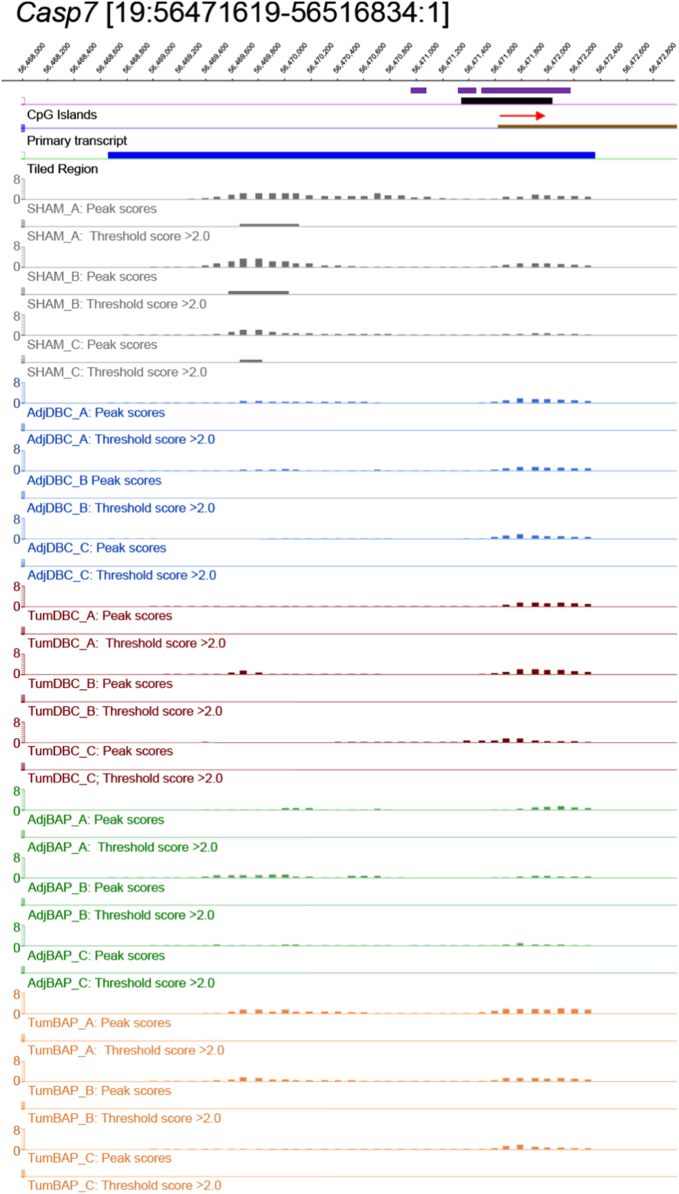
Methylation of *Casp7* promoter in normal and tumor lung tissues.

**Fig. 9:**
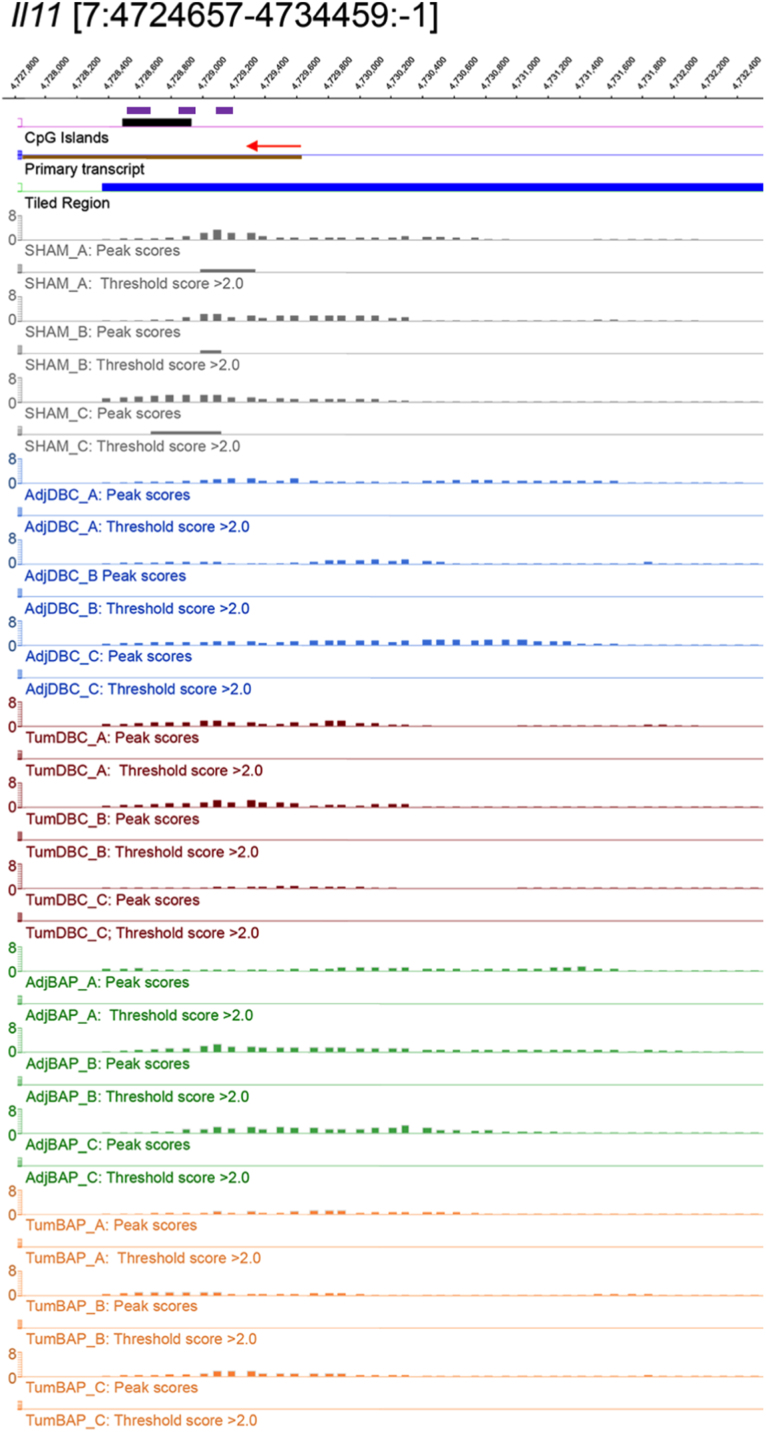
Methylation of *Il11* promoter in normal and tumor lung tissues.

**Fig. 10:**
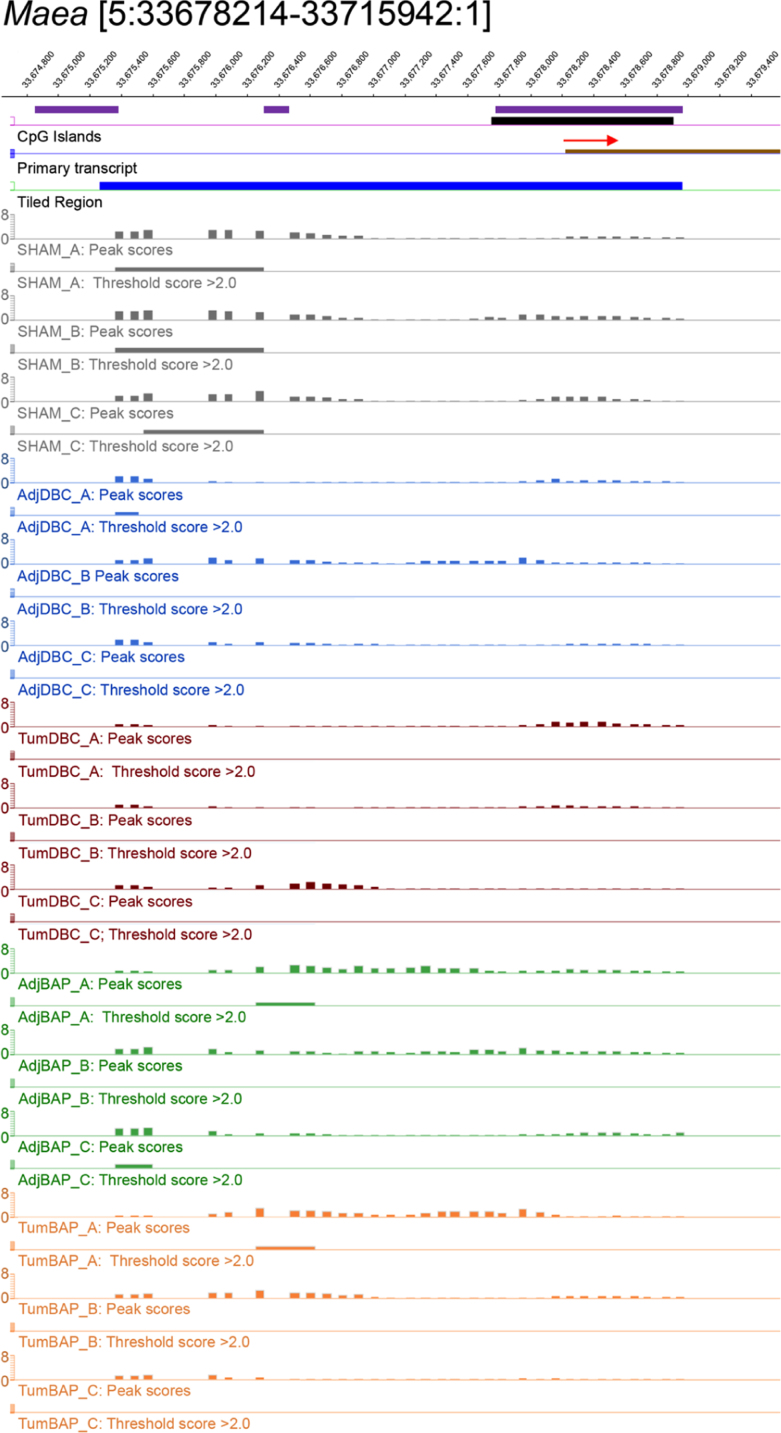
Methylation of *Maea* promoter in normal and tumor lung tissues.

**Fig. 11:**
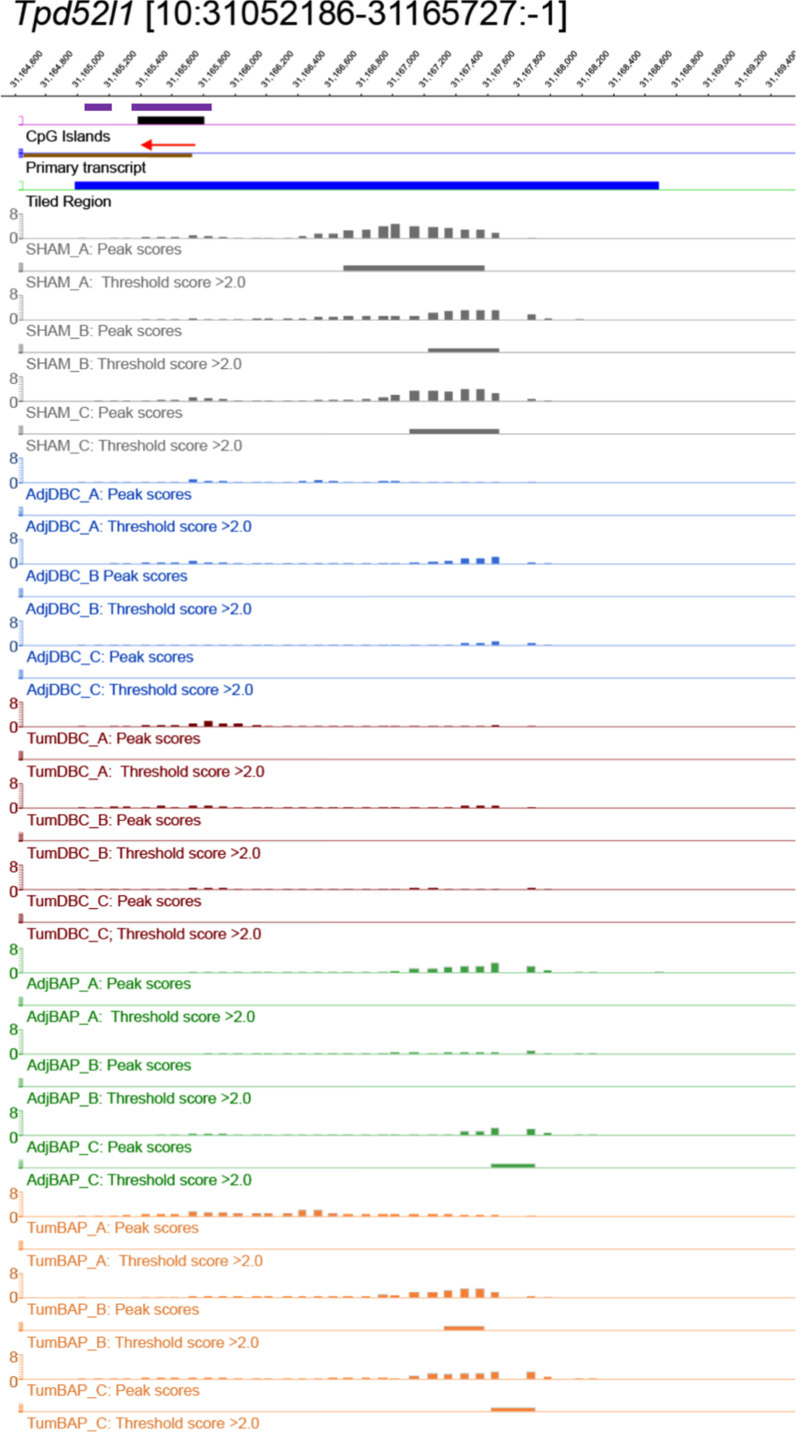
Methylation of *Tpd52l1* promoter in normal and tumor lung tissues.

For ten selected genes, Fig. 2, Fig. 3, Fig. 4, Fig. 5, Fig. 6, Fig. 7, Fig. 8, Fig. 9, Fig. 10, Fig. 11 depict methylation profiles aligned with predicted CpG islands across all tissue types. Each figure illustrates methylation profiles for the promoter region of the indicated gene obtained using the NimbleGen Mouse DNA Methylation 3×720K CpG Island Plus RefSeq Promoter Array. Chromosomal location, predicted CpG islands (black) and tiled regions are all mapped according to the NCBI37/mm9 mouse genome assembly. Also, CpG islands predicted by EMBOSS Cpgplot are shown in purple. Tracks representing control samples are shown in grey, adjacent normal DBC samples as blue, tumor DBC samples as red, adjacent normal BaP as green and tracks for tumor BaP as orange. Each sample is represented by two tracks, the peak scores and threshold score >2.0. The threshold score >2.0 track visualizes, by means of the solid bar, the region of peak scores that have surpassed the significance threshold and are thus considered to be hypermethylated. The peak score track displays the resulting score for each probe calculated as -log10 *p*-value using the one-sided Kolmogorov-Smirnov test.

## 2 Experimental design, materials and methods

### 2.1 Animal use and study design

The complete study protocol describing the source of carcinogens used, the husbandry and dosing of mice and the collection of samples is provided in detail in the accompanying paper [1]. Briefly, B6129SF1/J female and 129S1/SvImJ male mice were bred to obtain a backcross strain that is sensitive to DBC and BaP as transplacental carcinogens. Pregnant mice were dosed orally with either 3.75 mg/kd/day DBC, 12.5 mg/kg/day BaP or corn oil (sham) on gestation days 5, 9, 13 and 17. At age 45 weeks, offspring were humanely euthanized and their lungs removed for inspection of lung tumors. Normal lung tissues from sham-exposed offspring, lung tumor tissues from DBC- and BaP-initiated offspring, and normal tissues adjacent to tumor from DBC- and BaP-initiated offspring were collected and frozen for later use.

### 2.2 NimbleGen DNA methylation array

Complete methods for the isolation of genomic DNA from lung tissue samples, methylated DNA immunoprecipitation, whole genome amplification and array hybridization are provided in the accompanying paper [1]. Briefly, DNA was isolated from tissue samples using a standard purification kit (DNeasy blood and tissue kit, Qiagen), subject to restriction digestion to fragment the DNA and then purified again using the QIAquick PCR Purification kit (Qiagen). About 10–15 ng of purified genomic DNA was held in reserve as the control (input DNA), while the remainder was enriched for methylated DNA using the Methylated-DNA immunoprecipitation kit (Zymo Research). Both portions of DNA were subject to whole genome amplification, and then purified once again (QIAquick kit). Control and methylated DNA IP samples were sent to Roche NimbleGen for array hybridization and data quality control processing per their standard, validated protocols.

### 2.3 Genome-wide DNA methylation data processing

A complete description of methylation data processing and analyses performed is provided in the accompanying paper [1]. In brief, data received from NimbleGen׳s genome-wide DNA methylation analysis consisted of raw and processed data files for all samples. For each array feature, a scaled log_2_ ratio was calculated as the ratio of the input signals for the experimental and control samples co-hybridized to the array. Then, a one-sided Kolmogorov-Smirnov test was applied to identify probes with apparent high methylation within a 750 bp window, yielding a −log_10_ *p*-value. NimbleScan detected peaks by identifying at least two probes with a −log_10_ *p*-value>2, and peaks within 500 bp of each other were merged. Finally, the peak score was calculated as the average −log_10_ *p*-values from probes within that peak. Gene lists for clustering and gene ontology analyses were generated by selecting features with peak score>2 (indicative of methylated DNA) in all three samples from the treatment group of interest and by excluding all features that were not methylated (peak score<2) in at least two of the three samples from the comparison treatment group(s). Unsupervised, bi-directional hierarchical cluster analysis was performed using TM4 Multi-Experiment Viewer [9]. Gene ontology analyses were performed using the AgriGO SEA tool [6][1] against the mouse gene ontology database (Mouse Genome Informatics) with the following parameters: Fisher test with FDR under dependency correction and significance level of *P*<0.05 and the minimum number of mapping entries set at five genes. The gene ontology type performed was a generic GO slim (Gene Ontology Consortium).
